# Dissecting the Relationship between Root Morphological Traits and Yield Attributes in Diverse Rice Cultivars under Subtropical Condition

**DOI:** 10.3390/life12101519

**Published:** 2022-09-29

**Authors:** Md. Salahuddin Kaysar, Uttam Kumer Sarker, Sirajam Monira, Md. Alamgir Hossain, Md. Sabibul Haque, Uzzal Somaddar, Gopal Saha, Apurbo Kumar Chaki, Md. Romij Uddin

**Affiliations:** 1 Department of Agronomy, Bangladesh Agricultural University, Mymensingh 2202, Bangladesh; 2 Department of Crop Botany, Bangladesh Agricultural University, Mymensingh 2202, Bangladesh; 3 Department of Agronomy, Patuakhali Science and Technology University, Dumki, Patuakhali 8602, Bangladesh; 4 On Farm Research Division, Bangladesh Agricultural Research Institute, Gazipur 1701, Bangladesh; 5 School of Agriculture and Food Sciences, The University of Queensland, St. Lucia, QLD 4072, Australia

**Keywords:** root porosity, total dry matter, principal component analysis, correlation matrix, yield

## Abstract

Understanding the link between root morphological traits and yields is crucial for improving crop management. To evaluate this link, a pot experiment was conducted in the net house of the Department of Agronomy, Bangladesh Agricultural University, Mymensingh, Bangladesh during the *boro*(dry season irrigated) rice growing season of 2019–20. Thirteen cultivars, named BRRI dhan29, BRRI dhan58, BRRI dhan67, BRRI dhan74, BRRI dhan81, Binadhan-8, Binadhan-10, Hira-2, Tej gold, SL8H, Jagliboro, Rata boro, and Lakhai, were used following a completely randomized design (CRD) with three replications. The cultivars were screened for root number (RN), root length (RL), root volume (RV), root porosity (RP), leaf area index (LAI), total dry matter (TDM), and grain yield (GY). A considerable variation in root traits, LAI, and TDM were found among the studied cultivars, and the highest GY (26.26 g pot^−1^)was found for Binahan-10. Thirteen cultivars were grouped into three clusters using hierarchical cluster analysis, where clusters 1, 2, and 3 assembled with 3, 5, and 5 cultivars, respectively. Considering all of the studied traits, Cluster 3 (Binadhan-10, Hira-2, BRRI dhan29, BRRI dhan58, and Tejgold) showed promise, followed by Cluster 2 (BRRI dhan81, BRRI dhan67, SL8H, BRRI dhan74, and Binadhan-8). Principal component analysis (PCA) revealed that the RV, RDW, RFW, TDM, and GY are effective traits for rice cultivation.

## 1. Introduction

Rice (*Oryza sativa* L.),the staple food plant with the highest demand worldwide, provides daily sustenance to more than half of the global population. In 2020, around 497.69 m tons of rice was produced on 162.06 m ha of land around the world [1]. Meanwhile, approximately 37.61 million metric tons of rice is produced in Bangladesh from 11.72 m ha of land. *Boro* rice contributes 52.84% of the total rice production in Bangladesh and, in 2020, 74% of the total land area was occupied by rice cultivation [2].

The root system is described as the “hidden half”, because it performs a pivotal function in overall crop development as well as in advancement [3]. The root systems have long been considered to be a vital part of plants, as they supply nutrition; furthermore, they may also produce as well as transmit physiological catalysts [4,5]. Hence, the root systems play a crucial role in plant development, which subsequently regulates shoot development and seed yield [6,7,8,9], by serving as the “receptors” in a crop plant. The development and advancement of aboveground plants are closely related to root morphology [10,11,12]. Roots act as the supporting organ for resource transposition, metabolic activities, and the uptake of moisture and nutrients in the top half as well as the bottom half of rice plants [13,14,15]. Root characteristics influence the development and improvement of shoot parts via the reformed root-to-shoot distribution of nutrient elements or assorted naturally occurring signal transduction compounds, such as hormones, proteins, and RNAs [16].

There already exists research into the link between root attributes and crop output in rice, as it is a crucial component of rice production science [17,18,19]. The root biomass and root-to-shoot ratio at the tiller developing phase show no substantial correlation with tiller and panicle numbers, while rice grain yield and the root-to-shoot ratio at heading time and maturity showed a negative correlation with each other [20].

Robust roots may feed the shoots with adequate nutrition, water, and hormones, which can subsequently boost the growth of the aboveground parts of the plant [21]. Meanwhile, it has been asserted that roots are the site where mineral nutrients and moisture are absorbed; they also utilize photosynthates synthesized in the plant’s aboveground part to aid in root establishment and development. Due to the inherent competition between developing roots and shoots for available carbohydrates, roots with higher morpho-physiological attributes can utilize extra energy as well as carbohydrates, which restricts shoot development [19,22]. According to this assertion, the principles of “root growth redundancy” have been advocated: that is, an extremely extended root system would lead to unacceptable energy utilization, as well as becoming detrimental to the production of seed yields [20,23,24,25]. The plant roots primarily serve to absorb moisture and minerals from the rhizosphere around them [26]. Roots act as the “Engine” of rice development by absorbing the moisture and nutrients that are essential for plant development, and their phenotypic attributes have a strong association with the growth of shoots at the prophase stage, as well as with crop yields and seed qualities at advanced stages. Moreover, this serves as a baseline and ensures plant output maintenance for the superior phenotypic traits of roots [27]. Root development and root activities are assumed to have mutual interactions with shoots. A high photosynthetic rate of aboveground plant parts ensures high root activity by providing an adequate supply of assimilates to the root systems. In contrast, enhanced root activity ensures a high photosynthetic rate by providing shoots with enough nutrients, which subsequently results in greater yields and production rates [28].

Multivariate analysis is a method commonly adopted to assess the associations between a broad range of variables, and also to locate genotypic variability among complex data sets [29]. Principal component analysis (PCA) is an incredibly beneficial tool for dissecting the correlation between traits, as well as their interactions, and determining the genotypic performance in crop plants. A high-yielding rice variety might possess a root system that is pronounced as well as robust [30]. There are data available on root characteristics and yield in general, but no information on the interaction between root traits and yield in Bangladesh, and, in particular, for *boro* rice cultivars. Such insights are crucial for determining the root attributes that will facilitate the selection and breeding of high-yielding rice varieties. Therefore, this study aimed to screen diverse *boro* rice cultivars in Bangladesh based on root morphological characteristics, and to explore the comprehensive links between root and yield attributes. Therefore, it is advantageous to develop a detailed understanding of how root traits relate to the vegetative growth and grain yield indices of *boro* rice (inbred, hybrid, and local cultivars).

## 2. Materials and Methods

### 2.1. Plant Materials and Site

Thirteen *boro* rice cultivars were used for screening in this study. Among them, five cultivars were purchased from the Bangladesh Rice Research Institute (BRRI), two cultivars were purchased from the Bangladesh Institute of Nuclear Agriculture (BINA), and six cultivars were purchased from a local market. The details of these cultivars are presented in Table 1. The research was carried out in the *boro* (dry irrigated) season of 2019–2020 at the net house of the Agronomy Department, Bangladesh Agricultural University, Mymensingh, Bangladesh (latitude: 24°42′55″, longitude: 90°25′47″). The location under the non-calcareous dark gray floodplain soil under the Old Brahmaputra Floodplain agro-ecological zone (AEZ 9) [31].

### 2.2. Experimental Design and Crop Management

The pot experiment was set up using a completely randomized design with 13 different treatments (cultivars) and three replications. The number of pots for each replication was 39 (13 × 3). These 39 pots were placed side by side in each replication, maintaining a distance of 10 to 25 cm in between them. The soil was collected from the Agronomy Field Laboratory at depths of 0–20 cm and sun dried, crushed, and properly mixed. Then, 25 kg of soil was placed in each of the 30 L plastic pots (each with a diameter of 35 cm), and the pots were placed in the net house of the Agronomy Department of BAU, Mymensingh. The soil was a clay-loam-type with the following characteristics: pH–H_2_O 5.81, Ec (µs/cm) 138, organic carbon (%) 1.14, total N (%) 0.16, available P (ppm) 23.8, available K (ppm) 88.57, and available S (ppm) 58.98. Chemical fertilizers, such as urea, triple super phosphate (TSP), muriate of potash (MoP), gypsum, and zinc sulphate, were applied at rates of 8.14 g, 2.5 g, 3.25 g, 2.81 g, and 0.9 g pot^−1^, respectively. One-third of urea and full doses of the other fertilizers were applied during the final pot preparation. The remaining urea (5.43 g) was top dressed at 20 and 40 DAT, respectively. Seedlings (which had been developed in the seedbed) of the selected rice cultivars were transplanted into the pots at the age of 40 days. Each pot had three hills containing two seedlings hill^−1^. During transplanting, 4 cm of standing water was managed in each pot. The irrigation continued up to 15 days before harvesting. Weeds were occasionally seen during the early development period and were manually uprooted. No chemicals were applied as there were no infestations of insects or diseases.

### 2.3. Determination of Root Morphological Traits

Root morphological traits were determined at 80 DAT. Three plants of each cultivar were uprooted from each pot and the estimated values of different traits were averaged.

#### 2.3.1. Root Number

The rice plants were pulled up by digging deeply, close to the base, after watering. After being uprooted, the roots were rinsed under running tap water. The RN of every plant was counted manually.

#### 2.3.2. Root Length (cm)

Root length (RL) was assessed from the base of the plant to the tip of the main axis of the root using a centimeter scale, and the total value was estimated.

#### 2.3.3. Root Volume (cm^3^ hill^−1^)

The water displacement technique was used to record root volume (RV) [32], which was measured in cm^3^ hill^−1^.

#### 2.3.4. Root Porosity (%)

To maintain the original temperature, the preserved roots were sealed in airtight polybags and kept in water. The weight of the empty pycnometer vial was taken, as was the weight of the water-filled vial. The temperature of the water-filled vial was gauged. Tissue paper was used to gently blot the sample roots in order to transfer free water to the blotting paper. Using an analytical balance, the root weight was taken. The water-filled vial was then filled with the root. The sunken roots within the pycnometer vial were handled with a rinsed needle to release the air bubbles found within the vial. An analytical balance was employed to determine the weight of the water and intact fresh roots. The roots were then taken out of the vial and homogenized with a glass mortar and pestle. The whole homogenate was poured into the pycnometer and filled to its capacity. The weight was taken once the homogenate and pycnometer were brought to room temperature. Root porosity (RP) was estimated using the following formula [33]:

$$\%\;\text{porosity}\;=\frac{W_{\text{hr}\;+\;w}-W_{\text{fr}\;+\;w}}{W_{w}+W_{\text{fr}}-W_{\text{fr}\;+w}}$$

where W_hr+w_ = weight of homogenized roots and water-filled pycnometer vial, W_fr+w_= weight of fresh roots and water-filled pycnometer vial, W_w_ = weight of water-filled pycnometer vial, and W_fr_ = weight of fresh roots.

#### 2.3.5. Physiological Traits

After separating the leaf blades from the leaf sheaths, the leaf area (LA) was determined with the help of a leaf area meter (LI 3100, Licor, Inc., LincoIn, NE, USA). The leaf area index (LAI) was estimated as the ratio of LA to the ground area. The LAI, crop growth rate (CGR), relative growth rate (RGR), and net assimilation rate (NAR) were calculated following the standard formulae [34,35].

#### 2.3.6. Total Dry Matter (TDM)

First, the weight of the sheaths, leaves, and roots was measured after 72 h of drying in an oven at 65 °C. The TDM was then estimated by summing the shoot dry matter, comprising the leaf blade, leaf sheath, and root dry matter.

#### 2.3.7. Yield and Yield Components

Grain was deemed mature when 90% of the grains were ripened. At maturity, the plants were carefully pulled out from the pots. After separating the roots, plants from each pot were tied into separate bundles and tagged appropriately. Data for plant height (PH), number of total tiller hill^−1^ (TTH), number of effective tiller hill^−1^ (ETH), panicle number (PN), panicle length (PL), and number of grains panicle^−1^ (GpP) for each plant were recorded. For grain collection, plant samples were sun dried and, finally, grain yield (GY) as well as straw yield (SY) data were recorded, while keeping the moisture at 14%. The harvest index (HI) was determined by dividing the dry grain yield with the total shoot biomass (grain + straw) yield.

### 2.4. Statistical Analysis

The open-source statistical software R (v4.0.5) was used to conduct one-way analysis of variance (ANOVA) and multivariate analysis [36]. The multiple comparisons of treatment means were conducted using R Studio software. The mean values of all the attributes were standardized to develop a two-way (cultivars–traits) hierarchical clustering heatmap using the *Complex Heatmap* package of the R software. The optimum number of clusters was determined by the gap statistic method using the function *fviz_gap_stat*. The packages *ggplot2*, *corrplot*, *Factoextra*, and *Factomine R* from R were used to develop a correlation matrix and a PCA biplot.

## 3. Results

### 3.1. Root Morphological Traits, Total Dry Matter, and Leaf Area Index

Different root traits, TDM, and LAI at 80 DAT varied significantly among the cultivars (Table 2). The root number ranged from 193.66 (Jagliboro) to 340.66 (Binadhan-10) among the cultivars. The RL differed substantially among the cultivars and the highest and lowest values were documented in Binadhan-10 (1476.16 cm) and Jagliboro (1426.00 cm), respectively. In the case of RV, Binadhan-10 gave the highest value (8.15 cm^3^ hill^−1^), followed by Hira-2 (8.08 cm^3^ hill^−1^) and BRRI dhan29 (8.01 cm^3^ hill^−1^), while Jagliboro gave the lowest value (5.90 cm^3^ hill^−1^). The value of RP varied significantly among the cultivars and ranged from 14.55% (Jagliboro) to 22.18% (Binadhan-10). With progressive plant growth stages, TDM increased significantly at different DAT points, irrespective of the cultivars. The highest TDM was recorded in Binadhan-10 (24.12 g plant^−1^), followed by Hira-2 (23.02 g plant^−1^) and BRRI dhan29 (22.72 g plant^−1^), while Jagliboro (15.98 g plant^−1^) produced the lowest value. During the growth stage, the LAI of different cultivars showed significant differences. The maximum LAI was found in Binadhan-10 (4.82), followed by Hira-2 (4.81) and BRRI dhan29 (4.80). The lowest LAI was recorded in Jagliboro (4.64).

### 3.2. Growth Parameters

**Figure 1:**
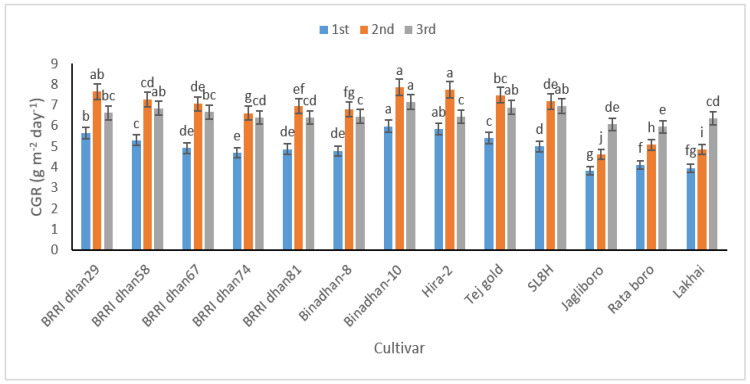
Crop growth rate (CGR) in 13 rice cultivars at 20–40 (1st), 40–60 (2nd), and 60–80 (3rd) DAT. In figure, same letter(s) do not differ significantly whereas dissimilar letter differ significantly.

The increase in plant weight per unit of area covered by the plant over a predetermined period is known as the crop growth rate (CGR). As leaf area increased over time, it was likewise noted that CGR increased until 60 DAT and then declined at 80 DAT in all cultivars except Jagliboro, Rata boro and Lakhai (Figure 1). At 20–40 (1st), 40–60 (2nd), and 60–80 (3rd) DAT, Binadhan-10 produced maximum CGR values of 5.99, 7.88, and 7.15 g cm^−2^ day^−1^ respectively, while the cultivar Jagliboro had the minimum CGR values were 3.84, 4.6, and 6.07 g cm^−2^ day^−1^, respectively.

**Figure 2:**
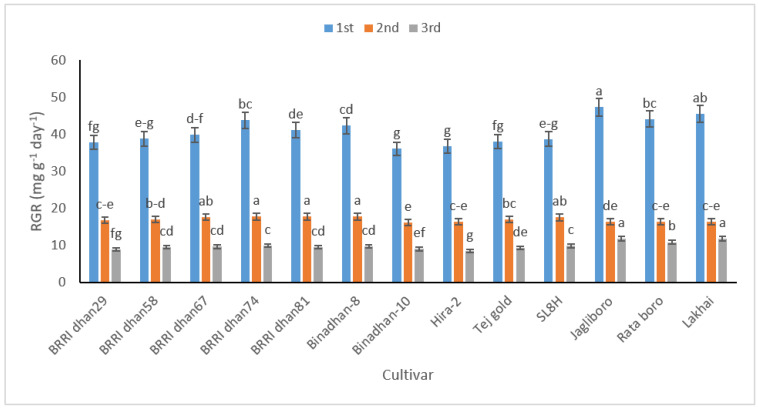
The relative growth rate (RGR) in 13 rice cultivars at 20–40 (1st), 40–60 (2nd), and 60–80 (3rd) DAT. In figure, same letter(s) do not differ significantly whereas dissimilar letter differ significantly.

RGR was higher in the early stages and began to decline as the plants got older (Figure 2). The rise in metabolically active tissue, which makes a smaller contribution to plant growth, is likely what caused the reduction in RGR. At 20–40 (1st) DAT, Jagliboro had the highest RGR (47.33 mg g^−1^ day^−1^) and Binadhan-10 had the lowest RGR (36.10 mg g^−1^ day^−1^). The maximum RGR value was documented in BRRI dhan74 (17.79 mg g^−1^ day^−1^), while the minimum value was reported in Binadhan-10 at 40–60 (2nd) DAT (16.27 mg g^−1^ day^−1^).Finally, between 60 and 80 (3rd) DAT, Lakhai had the highest RGR (11.79 mg g^−1^ day^−1^), while Hira-2 had the lowest RGR (8.54 mg g^−1^ day^−1^).

**Figure 3:**
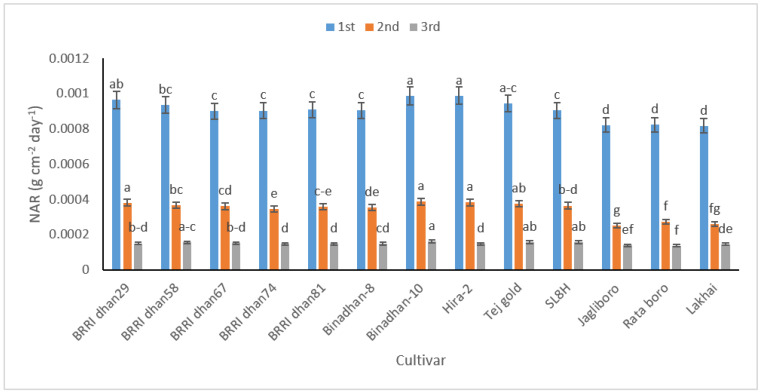
The net assimilation rate (NAR) in 13 rice cultivars at 20–40 (1st), 40–60 (2nd), and 60–80 (3rd) DAT. In figure, same letter(s) do not differ significantly whereas dissimilar letter differ significantly.

The net assimilation rate (NAR) refers to the amount of dry matter synthesized per unit of LA per unit of time. This implies that the synthesis of assimilation is driven by factors such as leaves and light. The wider the leaves and the more light they can absorb, the more assimilation will be detected. NAR increases if all the leaves block the light and are not shaded. This indicates that, while the LAI produced is high, the shade of the underlying canopy reduces the number of leaves that can be blocked, resulting in a decrease in NAR. In our study, the trends in NAR were more or less consistent, and decreased for all cultivars (Figure 3).

### 3.3. Yield Attributing Characters and Yield

The yield components and yields of the different cultivars are presented in Table 3. The highest ETH (14.66) was recorded in Binadhan-10, while the lowest (8.33) was observed in Jagliboro. The values of PL forHira-2 (24.78 cm) and BRRI dhan29 (23.65 cm) were the next highest, after Binadhan-10 (25.01). The values of GpP ranged from 89.66 to 125.00. The highest GpP (125.00) was observed in Binadhan-10, followed by Hira-2 (122.66) and BRRI dhan29 (122.00), whereas the lowest (89.66) was noted in Jagliboro. The 1000-grain weight ranged from 17.91 g (Jagliboro) to 26.49 g (Binadhan-10). The GY ranged from 17.28 to 26.26 g pot^−1^. Binadhan-10 produced the maximum GY (26.26 g pot^−1^), followed by Hira-2 (25.78 g pot^−1^) and BRRI dhan29 (24.22 g pot^−1^), whereas Jagliboro produced the lowest GY (17.28 g pot^−1^). The SY ranged from 17.72 to 26.57 g pot^−1^. The maximum SY (26.57 g pot^−1^) was recorded in Binadhan-10, followed by Hira-2 (26.21 g pot^−1^) and BRRI dhan29 (24.38 g pot^−1^); meanwhile, the lowest (17.72 g pot^−1^) was recorded in Jagliboro. The highest BY (52.83 g pot^−1^) was recorded in Binadhan-10, while the lowest (35.01 g pot^−1^) was found in Jagliboro. Variety had no significant influence on HI. BRRI dhan29 had the maximum HI (49.80 %), while Jagliboro had the lowest (49.36%) HI.

### 3.4. Cluster Analysis of Cultivars and Traits

**Figure 4:**
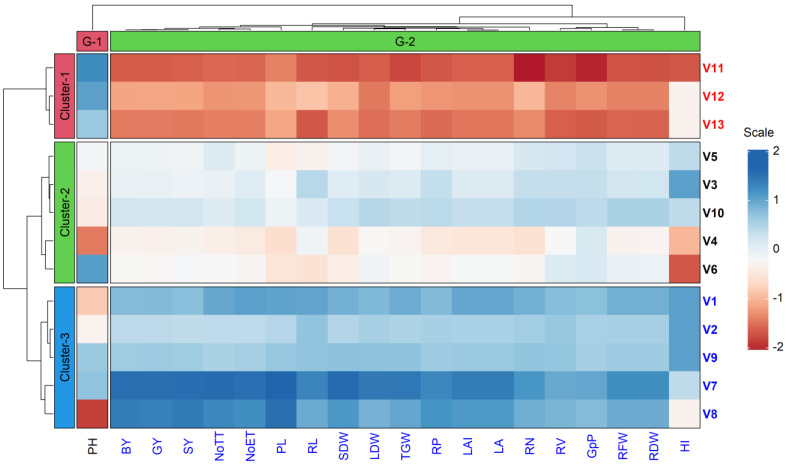
Heatmap with dendrogram (two-way; row and column) illustrating the groupings of the studied cultivars and measured traits. The 13 rice cultivars were divided into three (row) clusters, while 20 measurable attributes were sorted into two (column) groups. The heatmap was generated using the normalized mean values of traits (scale range: −2 to +2). Trait details: PH—plant height (cm); BY—biological yield (g pot^−1^), GY—grain yield (g pot^−1^); SY—straw yield (g pot^−1^); NoTT—number of total tillers; NoET—number of effective tillers; PL—panicle length (cm); RL—root length (cm); SDW—shoot dry weight (g plant^−1^); LDW—leaf dry weight (g plant^−1^); TGW—1000-grain weight (g); RP—root porosity (%); LAI—leaf area index; LA—leaf area; RN—root number; RV—root volume (cm^3^ hill^−1^); GpP—grain panicle^−1^; RFW—root fresh weight (g plant^−1^); RDW—root dry weight (g plant^−1^); HI—harvest index (%).

The cluster analysis, as shown in Figure 4, includes yield attributes and yield, root traits, and their respective contributions to the overall genetic diversity. Thirteen rice cultivars’ Euclidean distances were calculated with the help of normalized phenotypic parameters/standardized morphological data, and a two-way hierarchical clustering heatmap (cultivars and traits; row and column), along with a dendrogram, is shown in Figure 4. Based on the divergence in traits, the 13 rice cultivars were grouped into three row clusters (Figure 4). Cluster 1 had three (3) cultivars that were closely linked to one another, whereas Cluster 2 and Cluster 3 are each composed of five (5) cultivars. The assessed 20 traits, however, were divided into two column clusters comprising 1 trait for Group1 and 19 traits for Group 2. The trait PH was placed in Group1. All the root traits and yield-contributing traits, such as RV, RL, RN, RP, LAI, RFW, RDW, SDW, LDW, NoTT, NoET, TGW, and GpP were placed in trait Group 2. Cluster 3, comprising the cultivars Binadhan-10, Hira-2, BRRI dhan29, BRRI dhan58, and Tejgold, performed better in that it achieved greater standardized values of the measured traits and their outcomes followed by Cluster 2 (BRRI dhan81, BRRI dhan67, SL8H, BRRI dhan74, and Binadhan-8) and Cluster 1(Rata boro, Jagliboro, and Lakhai). The cultivars belonging to Cluster 1 exhibited poor performance in comparison to the cultivars of the other clusters. The cultivars Binadhan-10 and Hira-2 in Cluster 3 outperformed the other studied cultivars, showing higher normalized values of all traits (Figure 4).

### 3.5. Principal Component Analysis (PCA)

**Figure 5:**
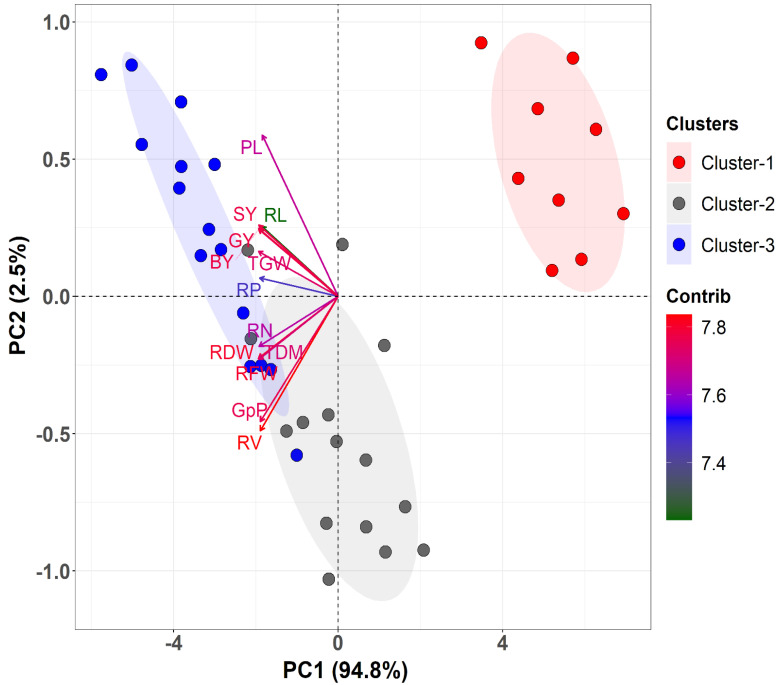
Principal component analysis (PCA) biplot depicting the relationship between the estimated variables and the rice cultivars.PC1 on the *x*-axis accounted for 94.8% of the total variability, while PC2 explained 2.5% of total variability and is shown on the*y*-axis. The different colors of the individuals (cultivars) represent the three clusters of 13 rice cultivars. The contribution of traits to PC1 and PC2 is indicated by the length and color intensity of the arrows. The darker red and longer arrows denote a higher contribution, whereas the darker green and shorter arrows denote the variables with lower contributions. RV—root volume (cm^3^ hill^−1^); RL—root length (cm); RN—root number; RFW—root fresh weight (g plant^−1^); RDW—root dry weight (g plant^−1^); RP—root porosity (%); TGW—1000-grain weight (g); TDM—total dry matter (g plant^−1^); PL—panicle length (cm); GpP—grain panicle^−1^; SY—straw yield (g pot^−1^); GY—grain yield (g pot^−1^); BY—biological yield (g pot^−1^).

The principal component analysis (PCA) was undertaken in order to evaluate the extent of the variation between the genotypes and their relationships with the observed traits. The PCA revealed that the first two principal components (PCs), which had Eigen values ˃1, accounted for 97.3% of the total variation. The first and second PCs contributed to 94.8% and 2.5% of the overall divergence, respectively, and, therefore, a PCA biplot was developed using only the first two components (Figure 5). The PC1 comprised principally RDW, RFW, TGW, BY, GY, SY, TDM, and RN, while the PC2 was mainly associated with the traits PL, RV, and GpP. The three clusters of cultivars were clearly separated based on these traits. Among the traits studied, the RV, RDW, RFW, GY, SY, and BY were shown to be highly contributing attributes (Figure 5).

### 3.6. Correlation of Root and Morphological Traits with Yield

**Figure 6:**
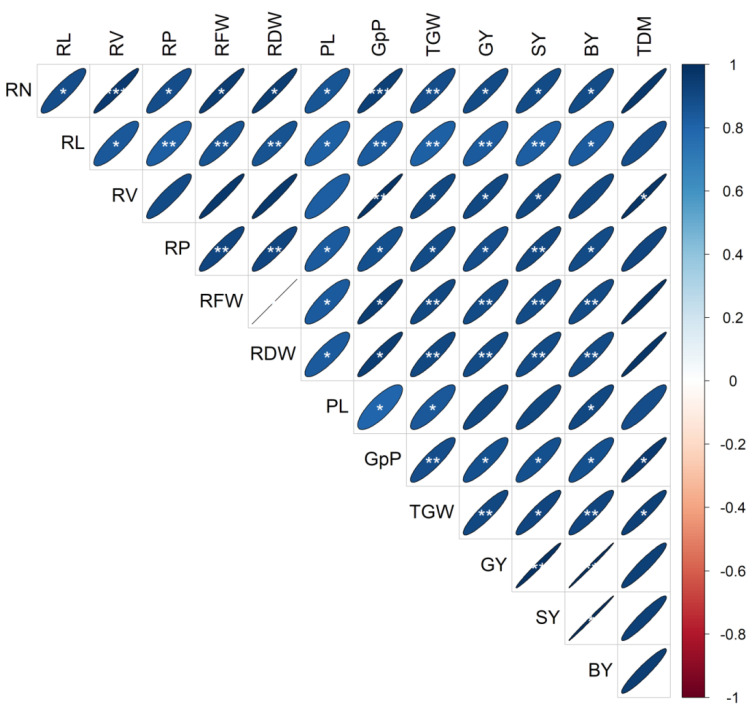
Correlation matrix and heatmap of the 13 assessed traits of the 13 rice cultivars. The positive and negative correlations are indicated by blue and red ellipses. A greater coefficient is reflected by a color of higher intensity. The degree of linearity of the relationships is highlighted by the shape of the ellipses. *, **, and *** denote 5%, 1%, and 0.1% levels of significance, respectively. Trait details: RV—root volume (cm^3^ hill^−1^); RL—root length (cm); RN—root number; RFW—root fresh weight (g plant^−1^); RDW—root dry weight (g plant^−1^); RP—root porosity (%); TGW—1000-grain weight (g); TDM—total dry matter (g plant^−1^); PL—panicle length (cm);GpP—grain panicle^−1^; SY—straw yield (g pot^−1^); GY—grain yield (g pot^−1^); BY—biological yield (g pot^−1^).

The correlation matrix of the 13 observed traits is displayed in Figure 6, to enable us to explore the associationsbetweenthem.GY showed a highly substantial positive association with RN, RL, RV, RP, RFW, RPW, GpP, and TGW. The RN and RL values were positively and significantly associated with all the traits, except TDM. However, only RV showed a significantly positive connection with TDM among the root traits. The results showed that root morphological traits had a positive and significant association with yield attributes and grain yield.

**Figure 7:**
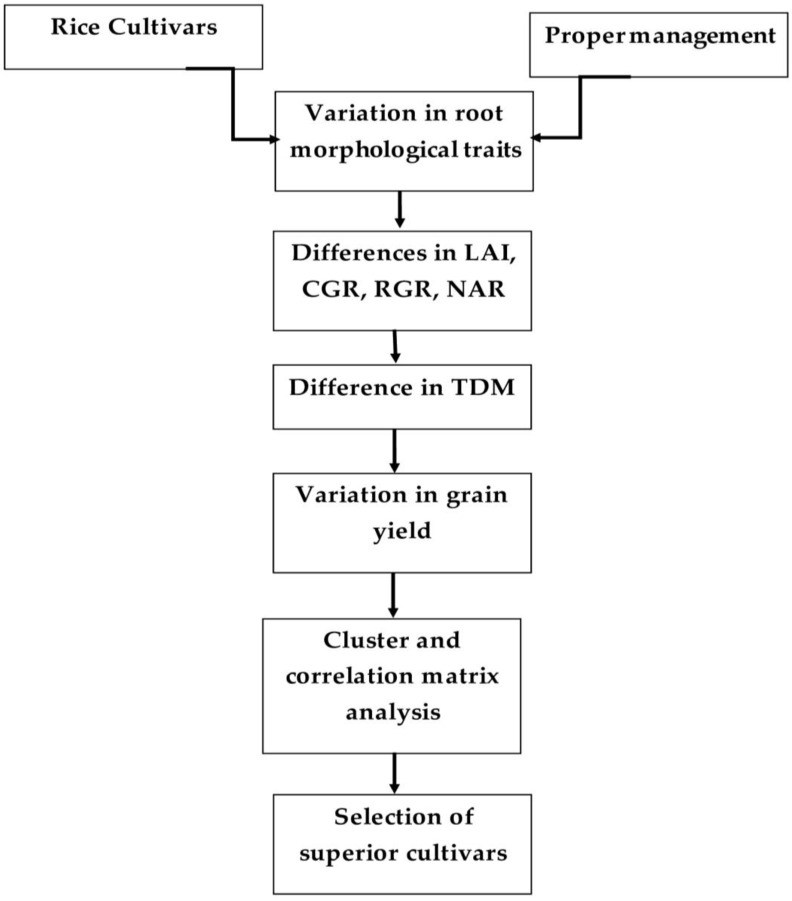
Mechanism involved in the selection of rice cultivars based on the relationship between root traits and yield.

Figure 7 illustrates the mechanism involved in the selection of rice cultivars depending on the relationship between root traits and yield. Root traits of the diverse rice cultivars varied significantly with proper management. Furthermore, variation also occurred in the rice physiological parameters (LAI, CGR, RGR, and NAR) and TDM. The yield attributes, including GP, PL, and TGW, also differed, which ultimately produced yield differences among the cultivars. Cluster and co-relation matrix analysis determined specific groups of cultivars and the relationships between root traits and yield parameters, respectively. Finally, considering the mentioned traits and their associations, Binadhan-10, Hira-2, and BRRI dhan29 were considered to be superior cultivars compared to the others.

## 4. Discussion

A robust root system increases a plant’s ability to absorb nutrients, boosting grain production [14]. Rice roots had an impact on the plant growth and grain yield, which was highly connected to root dispersion as well as to root morphology [21]. It has been reported that the uppermost layer (0–20 cm) of rice roots is crucial for moisture and nutrient absorption, which could improve the rate of grain filling, and, subsequently, the grain weight [37]. This study showed that total RN hill^−1^, RV hill^−1^, RL hill^−1^, and RP varied significantly among the cultivars.

The cultivar Binadhan-10 outperformed all the other cultivars in terms of root morphological features and yield, followed by Hira-2 and BRRI dhan29. Differences in root traits, growth parameters, and yield also occurred among the other cultivars. The genetic heterogeneity that exists among the cultivars may be the cause of the variations in root traits that have been reported. Genotypic differences in root traits have been documented in rice [38,39,40]. This study revealed that root traits were significantly correlated with yield, and might all perform a significant role in determining the selection of the best rice cultivar. Most studies demonstrate a strong association between rice root traits and the yield in the late growth stage [41,42,43].

An essential and effective agronomic indicator is the LAI, which measures crop growth and forecasts yield. LAI exerts a significant effect ton crop outputs [44]. In our study, the maximum LAI was recorded at 80 DAT, irrespective of the cultivar, and then it declined (data not shown). When spikelets started to grow, there was a drop in LAI dynamics. This was primarily because of leaf senescence; however, it is also likely that reserves were transferred to the expanding panicle. The former explanation emphasizes the significance of LAI and its connection to yields [45], which is corroborated by the positive association between LAI and yield. Researchers have noted a higher correlation between yield and LAI, indicating that yield rises as LAI grows [46,47].

The amount of carbohydrates deposited in plants prior to heading, and those produced by photosynthesis following heading, are the key determinants of grain production, which is a result of dry-matter buildup [48]. TDM varied significantly among the cultivars during various growth phases in this study. The cultivar Binadhan-10 performed better in terms of TDM at 80 DAT, followed by the cultivars Hira-2 and BRRI dhan29. Total dry matter accumulation varied significantly, and ranged from 19.88 to 21.99 g plant^−1^ depending on the genotype [49]. The cultivars Binadhan-10, Hira-2, and BRRI dhan29 may take up more nutrients from soil, which improves growth, increases LAI, and results in higher TDM. High-yielding cereals make the best photosynthetic progress during the vegetative growth stage and have an increased rate of dry matter production during the reproductive phase, which generally relies on an optimum LAI [50].

The tested cultivars varied significantly in relation to trends in growth attributes, namely CGR, RGR, and NAR. The sinus sheet plays a key role in photosynthesis and increases dry-matter content per unit area. In accordance with the findings of this study, cultivars with a larger LAI can be expected to have more CGR. In this study, the highest RGR was recorded in the early stages of plant growth, which linearly declined with time due to the loss of juvenility. At higher LAIs, increased shading leads to a decline in NAR. The cause of the significant decrease in NAR was possibly accelerated by leaf production and premature closing of the canopy. This is because, in this state, the leaves absorb less solar radiation and the NAR is decreased.

In recent years, the connection between root traits and grain yield has become a hot topic in rice research [21]. Even now, discussions continue regarding the question of which morphological attributes of roots might be responsible for a high-yielding rice cultivar. There is debate surrounding the matter of whether a higher root biomass and greater root activity might result in greater grain output in rice [19,21]. Previous research demonstrated a strong correlation between rice root number and root weight and grain yield [51,52,53]. The theory of “root redundant growth” posits that, due to significant energy expenditure, extensive root biomass or a high root–shoot ratio negatively impacts grain output [54]. In our study, we found that the RN hill^−1^, total RL hill^−1^, RV hill^−1^, and RP of 13 rice cultivars differed significantly, and revealed a positive correlation (Figure 6) with grain yield; no redundant root development was detected throughout this trial. Prior research explained that eliminating certain roots could promote rice development and boost grain yields, since redundant root development was evident when the supply of certain resources, such as water or nutrients, was restricted [20,25,55]. Our study confirmed that the absence of redundant root growth would result from sufficient nutrient uptake and optimum light harvesting by the cultivars and, therefore, displayed a significant positive relationship between root morphological attributes and grain yield.

As shown in Figure 4, the 13 studied cultivars were divided into three clusters based on genetic divergence analysis. The superiority among them was connected to the clustering of the cultivars. A completely randomized and independent clustering occurred, with Cluster 3 emerging as the superior cluster, followed by Cluster 2 and Cluster 1. Cluster 3, which comprised Binadhan-10, Hira-2, and BRRI dhan29, possessed higher yields and superior to root traits and yield components; we therefore recommend the cultivation of these cultivars in the *boro* dry season. Therefore, from the divergence analysis of the thirteen cultivars, the top three cultivars can be ranked as Binadhan-10 ˃ Hira-2 ˃ BRRI dhan29, based on the root traits, growth parameters, yield, and yield-contributing parameters. In African rice studies, cluster analysis revealed distinct groupings for each attribute based on the genotypes and surroundings. In the biplots, there seemed to be three unique habitats for yield and yield components [56]. According to hierarchical cluster analysis, lowland varieties tended to cluster in a shallow-angled group, whereas upland varieties tended to cluster in a steep-angled group [57].

The PCA showed the variance as well as the respective contributions of the assessed traits to overall heterogeneity (Figure 5). PCA biplots are widely used to determine important and effective attributes for selection. In this research, PC1 and PC2 contributed 94.8% and 2.5% of total variability, respectively. The PCA highlighted clear differentiation among the measured traits and substantial variability among the three clusters of 13 rice cultivars. The PCA biplot revealed that root attributes such as RDW, RV, RFW, and RN, as well as yield-related traits including TDM, SY and GY made significant contributions to both PCs. Thus, these attributes could effectively be used as selection criteria for the genetic improvement of rice cultivars. The first three PCs explained 90% of the variance in root traits among the 17 rice genotypes, demonstrating the remarkable consistency of the variable selection [58]. The first two PCs contributed to the majority of the variance in the initial dataset, describing 44.21% and 27.85%, respectively, of the variance. Together, they accounted for 72% of the variance. Additionally, 17.92% of the variance was accounted for by the third component (PC-3). The genetic similarities, as well as the genetic variability, among 114 rice accessions, were found in North East India [59]. The first major component (Eigenvalue—5.21) contributed 40.13% to the population’s overall variability, whereas a second component (Eigenvalue—2.0) contributed 15.38% to the overall variation. Fresh shoot weight, RV, dry shoot weight, and fresh root weight are two of these main components.

There was positive association between yield and HI, grain weight plant^−1^, and GpP [60]. Additionally, grain number, the main component of the sink size, has a positive relationship with GY [61]. In this research, the maximum GY of the top ranked cultivars, such as Binadhan-10, Hira-2, and BRRI dhan29, might be associated with more ETH and higher numbers of GpP. The findings also imply that the final yield performance has a significant impact on the biomass accumulated at 80 DAT. The dry matter accumulated in the culms and leaf sheaths would obviously be transferred into growing grains throughout the grain-filling phase. The physiological processes in the shoot can benefit from improved root morphology and physiology [62]. Greater yields and dry matter accumulations were influenced by the more highly rated morphological characteristics of roots [63]. In this study, Binadhan-10 had the highest value of all root traits and also the highest values for yield-contributing parameters, which is consistent with this statement. The interconnections between root traits and yield-related components conclusively prove the importance of RN, RL, RV, RP, and TDM.

## 5. Conclusions

Our results demonstrated a significant variation in root morphological traits among 13 rice cultivars, as well as a significant and positive association between root traits and yield. These results showed that boosting rice yields requires improved root properties. Hierarchical clustering based on the measured traits grouped the cultivars into three clusters, the cultivars Binadhan-10, Hira-2 and BRRI dhan29 in Cluster 3 performed better than the other cultivars. PCA indicated that the root volume, root number, root biomass, and grain yield are important and effective traits for selection criteria in rice breeding. Most yield-related variables have substantial positive correlations with root traits, indicating some useful synergies for selecting for high yields in rice cultivars.

## Figures and Tables

**Table 1:** List of the 13 rice cultivars, with their genetic origins and sources that were used in this study for screening of rooting patterns assimilate partitioning, and yield.

Sl. No	Cultivar	Genetic Origin	Parental Source/Accession Number	Source
1.	BRRI dhan29	Inbred	BG90-2 x BR51-46-5	BRRI
2.	BRRI dhan58	Inbred	Somaclone of BRRI dhan29 (Tissue culture)	BRRI
3.	BRRI dhan67	Inbred	BR61247-3B-8-2-1 x BRRI dhan36	BRRI
4.	BRRI dhan74	Inbred	BRRI dhan29 x IR68144	BRRI
5.	BRRI dhan81	Inbred	Amol-3 x BRRI dhan28	BRRI
6.	Binadhan-8	Inbred	IR29 x Pokkali	BINA
7.	Binadhan-10	Inbred	IR42598-B-B-B-B-12 x Nona Bokra	BINA
8.	Hira-2	Hybrid	-	Local market
9.	Tej gold	Hybrid	-	Local market
10.	SL8H	Hybrid	-	Local market
11.	Jagliboro	Local	8409	Farmer
12.	Rata boro	Local	8208	Farmer
13.	Lakhai	Local	4372	Farmer

**Table 2:** The root morphological traits, total dry mass (TDM), and leaf area index (LAI) of 13 *boro* rice cultivars at 80 DAT.

Cultivars	Root Number	Root Length (cm)	Root Volume (cm^3^ hill^−1^)	Root Porosity (%)	Total Dry Mass (g plant^−1^)	Leaf Area Index
BRRI dhan 29	330.33 b	1467.66 bc	8.01 bc	21.03 ab	22.72 bc	4.80 b
BRRI dhan58	315.33 d	1460.08 de	7.88 d	19.76 bc	21.97 d	4.77 d
BRRI dhan67	304.66 e	1454.83 fg	7.59 f	18.18 c	20.97 f	4.75 e
BRRI dhan74	262.33 h	1447.41 h	7.21 h	18.46 c	19.74 h	4.72 h
BRRIdhan81	288.00 f	1452.41 f–h	7.50 fg	19.30 bc	20.45 g	4.74 f
Binadhan-8	275.00 g	1450.83 gh	7.43 g	18.61 c	20.16 g	4.73 g
Binadhan-10	340.66 a	1476.16 a	8.15 a	22.18 a	24.12 a	4.82 a
Hira-2	332.00 b	1470.66 b	8.08 ab	22.02 a	23.02 b	4.81 ab
Tej gold	322.33 c	1463.50 cd	7.95 cd	19.96 bc	22.44 c	4.79 c
SL8H	308.00 e	1456.75 ef	7.75 e	19.45 bc	21.59 e	4.77 d
Jagliboro	193.66 k	1426.00 j	5.90 k	14.55 d	15.98 j	4.64 k
Rata boro	237.33 i	1437.33 i	6.31 i	15.94 d	16.84 i	4.68 i
Lakhai	227.66 j	1427.50 j	6.10 j	15.18 d	16.76 i	4.65 j
S$\bar{x}$	7.5	8.13	0.01	1.08	0.03	0.01
Level of sig.	**	**	**	**	**	**
CV (%)	0.95	0.19	0.76	5.53	0.90	0.12

Columns with treatment means that have similar letters do not vary significantly. ** = Significant at a 1% level of probability.

**Table 3:** Yield attributes and yields of rice as influenced by cultivars.

Cultivars	Effective Tillers Hill^−1^	Panicle Length (cm)	Grains Panicle^−1^ (no)	1000–Grain weight (g)	Grain yield (g Pot^−1^)	Straw Yield (g pot^−1^)	Biological Yield (g pot^−1^)	Harvest Index (%)
BRRI dhan29	13.66 a–c	23.65 ab	122.00 ab	25.11 ab	24.22 bc	24.38 b	48.60 b	49.80
BRRI dhan58	12.33 b–e	22.37 bc	119.66 bc	23.97 b–d	23.09 cd	23.28 bc	46.38 bc	49.79
BRRI dhan67	11.66 de	20.91 d	117.33 cd	22.94 cd	21.82 de	22.01 cd	43.83 cd	49.78
BRRI dhan74	10.66 e	19.97 d–f	115.00 d	21.90 d	20.98 e	21.41 d	42.39 d	49.51
BRRI dhan81	11.33 de	20.51 de	116.66 cd	22.23 d	21.65 de	21.89 cd	43.55 cd	49.71
Binadhan-8	11.00 de	20.21 d–f	115.00 d	22.05 d	21.22 e	21.70 cd	42.92 cd	49.43
Binadhan-10	14.66 a	25.01 a	125.00 a	26.49 a	26.26 a	26.57 a	52.83 a	49.69
Hira-2	14.00 ab	24.78 a	122.66 ab	25.26 ab	25.78 ab	26.21 a	52.00 a	49.58
Tej gold	12.66 b–d	22.83 b	120.00 bc	24.55 a–c	23.70 c	23.94 b	47.64 b	49.75
SL8H	12.00 c–e	21.14 cd	118.00 cd	23.65 b–d	22.54 c–e	22.85 b–d	45.40 b–d	49.66
Jagliboro	8.33 f	18.25 g	89.66 g	17.91 e	17.28 f	17.72 e	35.01 e	49.36
Rata boro	9.00 f	19.16 e–g	98.33 e	19.50 e	18.73 f	19.01 e	37.75 e	49.63
Lakhai	8.66 f	18.81 fg	94.00 f	18.89 e	17.88 f	18.18 e	36.07 e	49.58
S$\bar{x}$	0.84	0.61	3.92	1.26	1.01	0.89	3.47	0.42
Level of sig.	**	**	**	**	**	**	**	NS
CV (%)	7.97	3.68	1.74	4.95	4.58	4.24	4.21	1.31

Means that have the same letters within the same column do not vary significantly. ** = Significant at a 1% level of probability.

## Data Availability

Data sets analyzed in the present study are available from the relevant author on reasonable request.
